# Our New Name

**Published:** 1873-01

**Authors:** 


### PART III.

### EDITORIAL AND MISCELLANEOUS.

*Our New Name.*

We have the pleasure of greeting our readers with a new name. The GEORGIA MEDICAL COMPANION is no more. Its history is with the profession. Under a new name, our journal comes before them improved, we trust, in every particular. The SOUTHERN MEDICAL RECORD is the name it now bears.

In accordance with our request, and at the solicitation of friends, we invited our readers to suggest a new name for the journal. Many were sent in from nearly every Southern State. The name finally selected was suggested by Dr. R. C. Word, of Georgia, and we trust it will meet the approval of our friends everywhere.

The RECORD begins the new year under the most cheering and flattering prospects. Printed on good paper, with clear, new type, its mechanical execution cannot fail to please, while the utmost care and attention, as to its practical medical character for the future, is promised.

We again ask our friends to aid us, both by pen and personal effort, in extending the circulation of the RECORD. Its subscription list falls short now of few medical journals in the country. By the kindness and influence of its numerous friends, it can be made all they or the editors could desire.

